# Calling for a meaningful contribution? Bridging contributing to society with motivation theory

**DOI:** 10.3389/fpsyg.2023.1186547

**Published:** 2023-05-31

**Authors:** Rowdy Bryant, Evgenia I. Lysova, Svetlana N. Khapova

**Affiliations:** Department of Management and Organization, Vrije Universiteit Amsterdam, Amsterdam, Netherlands

**Keywords:** meaningful work, calling, contributing to society, Expectancy-Value Theory, fulfillment

## Abstract

This paper examines the concept of “contributing to society” in the context of meaningful work and calling. While previous studies have identified it as a significant dimension within these concepts, little attention has been paid to trying to conceptualize it. Also, with “self-oriented” fulfillment being an important aspect of the experience of meaningfulness, the understanding of contribution to society might be more complex than being simply an “other-oriented” concept. In response to this conceptual unclarity, we define contributing to society as a belief individuals hold about whether tasks positively impact work beneficiaries. We integrate this with Situated Expectancy-Value Theory (SEVT) to determine the expected task value of such belief. Our argument is that fulfillment of a contribution depends on three factors: (1) the expectation of a contribution based on someone's calling and expected meaningfulness; (2) the extent to which the employee is invested in the task, the costs of such task, whether the beneficiary and impact value and the utility for the self and beneficiary match the preference; (3) the extent to which this contribution is sufficient considering someone's expectation. Therefore, the expected task value can differ between individuals concerning the number and types of beneficiaries and the extent and value of the impact. Moreover, in this way contributions to society should also be perceived from a self-oriented perspective to be fulfilling. This original concept offers a theoretical framework and a research agenda that proposes new avenues of inquiry for calling, meaningful work, contributing to society, and related fields such as job design, and public policy.

## 1. Introduction

Many individuals want their work to matter, for it being significant and meaningful rather than merely a source of income (Dhingra et al., 2021). Therefore, having work that contributes to society–captured by the concepts of calling and meaningful work–is seen as one of the ways to do the work that matters (Wrzesniewski et al., 1997; e.g., Elangovan et al., 2010; Dik et al., 2012; Lips-Wiersma and Wright, 2012; Steger et al., 2012; Duffy et al., 2014; Martela and Pessi, 2018). When one's work contributes to society is seen as beneficial to both society and employees, this leads to work fulfillment and self-realization (Hackman and Oldham, 1975; Grant, 2007; Lepisto and Pratt, 2017; Smith, 2017; Bailey et al., 2019).

Despite the importance of contributing to society, the conceptual understanding of it has been lacking. What complicates this understanding is also a growing disagreement in whether meaningful work in itself is more self- (i.e., fulfilling) or other-oriented (i.e., contributing to society)^1^ (Bailey et al., 2019). In theory and in operationalization, both ways to experience meaningfulness have often been juxtaposed (see e.g., Ciulla, 2000, 2012; Wrzesniewski et al., 2003; Lips-Wiersma and Wright, 2012; Steger et al., 2012; Lepisto and Pratt, 2017; Michaelson, 2021). For example, while a contribution falls in the “other-oriented” dimension, seeking or finding oneself, and expressing one's full potential, apply to the “self-oriented” dimension (see Lips-Wiersma and Wright, 2012; Steger et al., 2012). Therefore, contributing to society is generally understood from the other-oriented dimension. However, more recent approaches have attempted to reconcile them, by stating that purposeful activity, such as work, is meaningful when it is both fulfilling and it contributes to others as employees can have person-dependent “good reasons” to consider something as meaningful (Wolf, 2010; Michaelson, 2021; Tyssedal, 2022). Accordingly, people's perception of what constitutes a valuable contribution can vary depending on their personal factors. However, as such conceptualizations have taken a philosophical point-of-view, we yet do not understand the process underlying the emergence of the experience of meaningful work in relation to this “other” that the contribution is oriented at Bailey et al. (2019).

In calling, scholars have also referred to “prosocial” or “other-orientation” as a separate dimension of its multidimensional construct (see e.g., Elangovan et al., 2010; Dik et al., 2012). With such dimensions, scholars typically refer to the orientation that employees have to contribute to others (see e.g., Steger et al., 2010; Shimizu et al., 2019). A similar discussion as in the meaningful work literature is taking place in the calling literature. For instance, scholars argue that self-oriented callings can have ethical value when it comes to employees' contributions (Michaelson and Tosti-Kharas, 2019). As such, self- and other-orientation might have more in common than previously thought. Yet, there is much unclarity on whether callings are more self-oriented, compared to being other-oriented, leading to conceptual issues (e.g., Both-Nwabuwe et al., 2017; Dik and Shimizu, 2019; Dobrow et al., 2019; Thompson and Bunderson, 2019). Hence, a better understanding of the dynamics between self- and other-orientation might shed light on not only a better understanding of what a contribution to society is, but also inform the calling and meaningful literature.

Motivation literature has suffered a similar discussion but has progressed by viewing self- and other-orientation as a dynamic process, instead of a dichotomy. Initially, self- and other-orientation were seen as two distinct dimensions (e.g., Korsgaard et al., 1996, 1997; De Dreu, 2006; De Dreu and Nauta, 2009), but more recent research suggests that they are a continuum (Murphy and Ackermann, 2014; Pfattheicher et al., 2022). This continuum allows individuals to progress toward a more self- or other-oriented approach, depending on their reasons to contribute to others (Butts et al., 2019; Zimmermann, 2020).

Integrating motivational theory with contributing to society^2^ has the potential to yield valuable insights. However, attempting to do so for all types of theories would be cumbersome. As employees can have “good reasons”, and that they may have a calling to contribute, implies that they have certain expectations regarding their contributions, we propose applying the Situated Expectancy Value Theory (SEVT)^3^ (Eccles-Parsons et al., 1983; Eccles et al., 1998). According to SEVT, individuals' motivation to engage in behavior is shaped by their anticipated task value, which reflects their perceived value of the desired outcome (Studer and Knecht, 2016). By focusing on SEVT, we can better understand the complex relationship between contributing to society, calling, and meaningful work.

The aim of this paper is to offer a deeper comprehension of the notion of contributing to society. To achieve this, we will initially define the concept by drawing on the literature of meaningful work and calling. Subsequently, we will utilize the SEVT to construct a theoretical framework that illuminates the correlation between self- and other-orientation in relation to contributing to society. Lastly, we will put forward a research agenda that explores how an improved understanding of contributing to society can advance these fields conceptually.

## 2. Theoretical background

In this paragraph, we will explore how calling and meaningful work have conceptualized contributing to society. Then, we will clarify our definition of the latter. Lastly, we will propose how SEVT can illuminate the dynamic interplay between the concepts.

### 2.1. Contributing to society: a “meaningful” perspective

Meaningful work is “work experienced as particularly significant and holding more positive meaning for individuals” (Rosso et al., 2010, p. 95). Formerly, it was accepted that employees generally work “to pay the bill” and not search for other sources of fulfillment (e.g., Wrzesniewski et al., 2003; Rosso et al., 2010). In the study of Wrzesniewski et al. (2003), the role of others was highlighted, as work meaning was defined as employees' understanding of what they do at work and the significance of their contributions. Sensemaking and significance were thus emphasized, as individuals cannot make sense of their work, or contributions, without considering that they matter to other people (Pratt and Ashforth, 2003; Bunderson and Thompson, 2009). Subsequently, scholars began operationalizing a subdimension of meaningful work as “greater good motivation” and “serving others”, emphasizing the positive difference that work can make in the world, such as to clients, organizations, human wellbeing, or the natural environment (Lips-Wiersma and Wright, 2012; Steger et al., 2012; Schnell et al., 2013). While contributing to society is less vivid in unidimensional measures, Both-Nwabuwe et al. (2017), after reviewing all operationalizations, opted to use the multidimensional scale as meaningful work obtains more conceptually different dimensions. This is, for instance, typified by the definition of Lips-Wiersma and Wright, who argued that meaningful work is “an individual, subjective and existential concept that is distinct from, but influenced by, organizational antecedents and outcomes” (Lips-Wiersma and Wright, 2012, p. 657). One of the important distinctions that was drawn based on this concept, was the idea that there are both self- and other-oriented dimensions of meaningfulness (Lips-Wiersma and Morris, 2009; Rosso et al., 2010; Lips-Wiersma and Wright, 2012; Steger et al., 2012; Martela and Riekki, 2018), with contributing to society falling within the other-oriented dimension.

Recent discussions on meaningful work have shifted toward a dichotomy between fulfillment and justification, aligning with contemporary theories in the field of meaning in life (Wolf, 2010). The subjective realm suggests that work is meaningful when people enjoy doing it or are achieving a career goal (Rosso et al., 2010; Lepisto and Pratt, 2017), whereas the justificatory realm suggests that work is meaningful when it contributes to others (Lepisto and Pratt, 2017; Michaelson, 2021). A recent study by Tyssedal (2022) highlights the different reasons that people use to determine whether something is worthy, such as whether working at a weapon factory is worthy. For instance, while one person might think that work in a weapon factory is immoral, someone else might find it valuable since it allows them to develop high-quality weapons actively used to defend the country. It is noteworthy that while numerous individuals may engage in acts of contribution toward others, the extent to which such contributions are deemed meaningful is contingent on the degree to which they align with an individual's personal preferences and values. In other words, the act of contributing only acquires a sense of meaning when it resonates with an individual's subjective disposition and inclination (e.g., Martela and Pessi, 2018; Lysova et al., 2019a).

Unfortunately, there are still certain paradoxes that directly relate to these contributions (see Bailey et al., 2019). For instance, meaningfulness stems from self-fulfillment and self-actualization, but its realization is contingent on others. This perspective prompts the question of how self- and other-oriented versions of meaningfulness interconnect. Do individuals experience meaning simply by contributing to any beneficiary that comprises “society”? Or do they have preferences? Additionally, do employees merely want to provide the beneficiary with *any* additional value, or does the value also need to hold significance for the individual?

### 2.2. Contributing to society: a “calling” perspective

Wrzesniewski et al. (1997) introduced the concept of calling, defined as “the experience of work as a fulfilling and socially useful activity” (p. 22), which has since garnered widespread recognition. Subsequent research has refined and expanded on this initial framework. For instance, Dik and Duffy (2009) argued that the origin of a calling can be external to the individual and take the form of a transcendent summons. Other scholars have focused on the role of religion in motivating individuals to contribute to the greater good (Steger et al., 2010; Hirschi, 2011), which has been described as a “neoclassical” understanding of calling (Bunderson and Thompson, 2009). In the past, calling was often associated with religious vocations, such as becoming a priest or a nun, where individuals felt a spiritual calling to dedicate their lives to serving God and their communities (see Christopherson, 1994; Davidson and Caddell, 1994). In this sense, job calling was seen as a divine mandate, and the idea was that individuals were fulfilling a higher purpose by using their talents and skills in service of others (Hall and Chandler, 2005; Thompson and Bunderson, 2019).

Over time, the idea of job calling has become more secularized, and it is no longer confined to religious vocations (Steger et al., 2010; Thompson and Bunderson, 2019). Today, many people view their careers to make a significant contribution to society or to pursue a personal passion or interest. Scholars promoting a more secular understanding of calling also discuss contribution to others as an aspect of calling but to a lesser extent or less vividly (Elangovan et al., 2010). The emphasis on religious motivations has thus given way to a broader focus on a prosocial, or other-orientation, to contribute to society (Hagmaier and Abele, 2012; Praskova et al., 2015; Zhang et al., 2015; Shimizu et al., 2019; Willner et al., 2020). This shift also meant that calling not only concerns more societal contributions, but also helping colleagues (e.g., Grant, 2007; Schott et al., 2019; Ritz et al., 2020). While the distinction between self- and other-oriented types of callings persists (Michaelson and Tosti-Kharas, 2019), the former dimension has been the subject of recent research that seeks to clarify the underlying mechanisms and implications of a calling. In this sense, job calling is less about a divine mandate and more about personal fulfillment and making a positive difference in the world. As such, the question is how we can, then, interpret calling from both a self-oriented (i.e., fulfillment) and other-oriented (i.e., contributing to others) perspective. Lysova et al. (2019b) called for research that offers new insights into the diverse (and debated) understandings of what calling means, and the implications of these differences. Therefore, do contributions to society differ as a matter of kind, or degree?

The concept of calling has evolved to encompass a more secular understanding of work that places greater emphasis on fulfillment (e.g., Dik and Shimizu, 2019; Thompson and Bunderson, 2019). However, it remains unclear whether employees are called to contribute to society at large or if they may have different callings for different beneficiaries. Additionally, it is unclear whether employees aim to make an impact on any beneficiary or if they have different callings with respect to the values they wish to deliver.

### 2.3. Defining contributing to society

After introducing contributing to society within the context of meaningful work and calling, we will clarify what we mean by it. Various terms have been used in the literature to describe this phenomenon (see Table 1), but they all share the same idea, which leads to the following definition:

“*the belief that one's work task should/is positively impact(ing) beneficiaries”*.

**Table 1:** Conceptualizations of contributing to society.

	**Meaningful work**	**Calling**
Dimension name	Significance (Bunderson and Thompson, 2009; Schnell et al., 2013) In service of something greater than the self (Rosso et al., 2010) Beneficence (Martela and Riekki, 2018) Broader purpose (Martela and Pessi, 2018) Greater good (Steger et al., 2012) Moral legitimacy (Bailey et al., 2019) Serving others (Lips-Wiersma and Morris, 2009; Lips-Wiersma and Wright, 2012)	Prosocial religiousness (Hirschi, 2011) Better world (Wrzesniewski et al., 1997) Work as religion (Steger et al., 2010) Altruism (Hunter et al., 2010; Zhang et al., 2015) Prosocial intention, orientation, duty (Dik and Duffy, 2009; Dik et al., 2012; Willner et al., 2020) Value-driven behavior (Hagmaier and Abele, 2012) Other-oriented (Praskova et al., 2015; Michaelson and Tosti-Kharas, 2019) Self-transcendent values (Shimizu et al., 2019)
Contribution to whom?	The world/humanity (Lips-Wiersma and Morris, 2009; Schnell et al., 2013) Society (Bunderson and Thompson, 2009; Steger et al., 2012; Schnell et al., 2013; Bailey and Madden, 2016) Human well-being (Lips-Wiersma and Wright, 2012) Natural environment (Lips-Wiersma and Wright, 2012; Martela and Pessi, 2018) Customers/clients (Lips-Wiersma and Wright, 2012; Martela and Pessi, 2018) Greater good (Steger et al., 2012) Others (Steger et al., 2012; Bailey and Madden, 2016; Martela and Riekki, 2018; Bailey et al., 2019) Community (Rosso et al., 2010) Family (Rosso et al., 2010; Martela and Pessi, 2018) Co-workers/organization (Rosso et al., 2010; Schnell et al., 2013) Societal challenges (Martela and Pessi, 2018; Bailey et al., 2019)	Natural environment (Elangovan et al., 2010) World (Wrzesniewski et al., 1997) Society (Hunter et al., 2010; Dik et al., 2012; Hagmaier and Abele, 2012; Zhang et al., 2015) Community (Hall and Chandler, 2005) Public health (Elangovan et al., 2010; Praskova et al., 2015) Greater good (Hirschi, 2011; Hagmaier and Abele, 2012; Steger et al., 2012; Duffy et al., 2014; Willner et al., 2020) Common good (Bailey et al., 2019) Others (Hunter et al., 2010; Dik et al., 2012; Michaelson and Tosti-Kharas, 2019)

These beneficiaries can be individuals, or groups, that are either closely related to the employee (e.g., clients or the organization), or more distantly related (e.g., the natural environment, the greater good, or the public) (also see Table 1).

It is important to note that this impact should be positive and in the interest of the receiving end, as harmful impact would not be considered a contribution (see e.g., Ciulla, 2012; Dik et al., 2012; Michaelson, 2021). The impact should also be achieved through purposeful work activities (Lysova et al., 2019a).

Furthermore, contributing to society is *subjective* and varies depending on the individual's *perception of the impact*. the notion of a “meaningful contribution” is subjective and varies from person to person. Individuals hold different attitudes toward certain values, some of which may be more or less normative depending on external factors such as cultural values or the influence of others (Michaelson, 2021). For instance, someone doing what is considered “dirty work” may believe that they are positively impacting clients, while others may view it as a meaningless contribution (e.g., Bunderson and Thompson, 2009; Bailey and Madden, 2016; Blustein et al., 2023). Therefore, callings and meaningful work differ between individuals—also depending on the (normative) reasons that someone has for engaging in contributing to society (see Michaelson, 2021; Tyssedal, 2022). As such, we would propose that contributing to society concerns the *belief* that work should (i.e., a “calling”) or is (i.e., “meaningful work”) positively impacting beneficiaries through one's work^4^. Which beneficiary is or will be impacted, will depend on the individual's belief of the importance of a certain beneficiary (Girschik et al., 2022).

However, for fulfillment to be achieved, certain *expectations* must be met, as not all contributions are meaningful or aligned with one's calling. While contributing to society and fulfillment have been traditionally considered separate dimensions, they may be theoretically viewed as the same dimension, with an employee's level of fulfillment dependent on their expectations of their contribution. To explore this idea, we will introduce the “Situated Expectancy-Value Theory” motivational framework.

## 3. A Situated Expectancy-Value Theory

### 3.1. Situated Expectancy-Value Theory

Expectancy-Value Theory (EVT) postulates that some choices are motivated by a combination of people's expectations for success and subjective task value (Eccles, 1994; Wigfield and Eccles, 2000; Eccles and Wigfield, 2002). The model further differentiates task value into four components: attainment value (i.e., the importance of doing well), intrinsic value (i.e., personal enjoyment), utility value (i.e., perceived usefulness), and cost (i.e., competition with other goals) (Leaper, 2011). The model was recently improved to add an S (situated) to the EVT acronym as empirical work in individuals' developmental histories, the socio-cultural beliefs and values that influence individuals as they develop, and the situations in which they find themselves, are of such importance (Eccles and Wigfield, 2023). Unlike its predecessors, in EVT expectancy refers to an individual's beliefs about the outcome of a particular activity. In other words, individuals will be more motivated to engage in something if they believe it will lead to a positive outcome. Therefore, this perspective fits with our definition of contributing to society in which certain expectations concerning the contributions apply.

While Situated Expectancy-Value Theory (SEVT) has been used in relation to educational achievement (Wigfield, 1994; Wigfield and Eccles, 2000; Rosenzweig et al., 2019; Ranellucci et al., 2020), it has been rarely used in the context of organizational and vocational behavior. Yet, the application of SEVT can be advantageous in comprehending the attitudes of employees toward work behavior deemed to be contributing to society as research shows that meaningful work and one's sense of calling are—to some degree—related and correlated (see e.g., Wrzesniewski et al., 1997; Duffy et al., 2012, 2013, 2014). As such, it is reasonable to study contributing to society by considering the expected outcomes of their work that contributes to others.

In the remainder of this paragraph, we will use the four dimensions (i.e., attainment value, intrinsic value, utility value, and cost) to better understand how contributing to society could be conceptualized through the lens of SEVT.

### 3.2. Attainment value

Attainment value reflects the *importance* individuals attach to participating in different tasks and activities and is based on personal and identity-related factors (Eccles-Parsons et al., 1983). This value is derived from the extent to which a task aligns with an individual's core self-schema, social and personal identities, and ought selves (Eccles and Wigfield, 2020, p. 14). The individual's self-schemata is important since this contains the perception that someone has of oneself, and lead to having goals, beliefs, and ultimately: expectations (e.g., Eccles-Parsons et al., 1983; Eccles and Wigfield, 2020).

#### 3.2.1. Self-schemata

The importance that individuals place on their contributions depends on their motivation or orientation. One important motivation could be having a calling, which means that the employee has set a (career) goal, or desire, to contribute to society (Dik et al., 2012; Michaelson and Tosti-Kharas, 2019), and could even see it as a duty (Bunderson and Thompson, 2009; Swen, 2020). However, personal and identity-related factors can vary across individuals. While some employees may value a calling highly, others may have more self-oriented motivations, such as meeting achievement needs (Wrzesniewski et al., 1997; Duffy and Sedlacek, 2011; Dik and Shimizu, 2019). The extent to which contributing to society will be important for the individual could, therefore, differ between and across individuals.

#### 3.2.2. Normative factors

Even employees with a self-oriented schema may deem contribute to society important, as contemporary organizations have CSR work roles or roles that benefit society (e.g., Evans and Davis, 2008). If organizations promote CSR, this could have effect on the employees as the issue becomes more pressing (Jones et al., 2019). Therefore, normative beliefs influence the importance of employees' contributions to society. The importance of contributing to society can also be influenced by normative beliefs that arise from societal expectations. For example, in some cultures, doing good for society is highly valued, while focusing solely on making money is not (Michaelson, 2021). Therefore, to form a vision of the common good, individuals need to have knowledge of what is appreciated in their society (Meynhardt et al., 2020). The relative importance of contributing to society varies across cultures, organizations, and individuals, and be influenced by the same factors (e.g., Lysova et al., 2019a). It will depend on the self-schemata, influenced by normative beliefs, that makes individuals place a different emphasis on self-orientation, (i.e., fulfilling a career objective) (Wrzesniewski et al., 1997; Dobrow and Tosti-Kharas, 2011), and other-orientation (i.e., the value for society) (e.g., Dik and Duffy, 2009; Rosso et al., 2010; Steger et al., 2012; Willner et al., 2020).

#### 3.2.3. Task preference and investment

Another important aspect is the purposefulness of work. Purposefulness is associated with beneficiary impact, and scholars have suggested that work activities are more meaningful when individuals are engaged in them (Bunderson and Thompson, 2009; Martela and Pessi, 2018; Lysova et al., 2019a; Michaelson, 2021). Individuals who value contributing to society are typically oriented toward finding a job that aligns with their values and are invested in their work tasks (Duffy and Dik, 2013). Thus, it is crucial for individuals' personal values to align with the values that a particular task can deliver. The level of investment in a task can determine an individual's level of dedication, which may lead to successful task completion.

### 3.3. Intrinsic value

Intrinsic value, as defined by Eccles-Parsons et al. (1983), encompasses the enjoyment one derives from a task. Similarly, like attainment value, an individual can find contributing to society important but can also find joy in performing them (Martela et al., 2018; Allan et al., 2019; Rothausen and Henderson, 2019).

#### 3.3.1. Expected fulfillment

Job satisfaction can also be found when the job is in alignment with the individual's calling, i.e., when the individual is living their calling (Duffy et al., 2012, 2013; Hagmaier and Abele, 2012). Therefore, the perceived outcomes of a job should, to some extent, align with the individual's self-schemata. Previously, it was widely accepted that fulfillment and contribution to others should be juxtaposed (see Lips-Wiersma and Wright, 2012; Steger et al., 2012; Lepisto and Pratt, 2017). Therefore, self-actualization and contribution to others become two necessary, separate, dimensions for the experience of meaningful work to occur. Also, in the calling literature it is widely accepted that other- and self-oriented dimensions are separate, yet both necessary for a calling to occur (e.g., Dik et al., 2012; Dik and Shimizu, 2019; Shimizu et al., 2019). As such, the two dimensions are connected in such way that, for instance, meaningfulness, involves both projects that one enjoys and that have a contribution to others (Wolf, 2010; Michaelson, 2021). However, something crucial is missing from this interpretation. While this perspective implies a connection between the activity and the outcome (i.e., contribution), it does not necessarily imply that the individual should enjoy the contribution itself. Or, as Tyssedal (2022) puts it, employees can have different reasons that establish someone's belief whether something will be meaningful. Therefore, it is crucial to better understand how employees come to understand their contribution in terms of the reason or expectations. Callings can help us understand how likely a person is to align their job with the meaning they attribute to their work and why they work (e.g., Wrzesniewski and Dutton, 2001; Peterson et al., 2009; Rosso et al., 2010; Scott-Morton and Podolny, 2012).

#### 3.3.2. Beneficiary and value matching

Although scholars in the field of calling and meaningful work have identified “contributing to society” as a significant dimension, the emphasis is often placed on generic terms such as “greater good” or “significant” without specifying the beneficiary. However, research has shown that individuals often have a clear preference for a specific beneficiary, such as healthcare patients or animals (Raatikainen, 1997; Bunderson and Thompson, 2009; Dobrow, 2013; Bailey and Madden, 2016; Schabram and Maitlis, 2017). When individuals contribute to a different beneficiary then their preferred choice, they may not find it as fulfilling. As contributing to society is a belief, individuals may not perceive their contribution as meaningful if it does not align with their preferences. Therefore, the focus on a generic terminology for contributing to society may overlook the importance of individual preferences and fulfillment.

#### 3.3.3. Preference vs. matching

Lysova et al. (2019a) argued that for meaningful work to occur, there should be fit with the individual, job, organization, and society. A similar fit is needed in the case of a contribution to society. The desired contribution, both in terms of value and beneficiary, can differentiate individuals based on their sense of calling and meaningfulness. When the job's task, value, and beneficiary are congruent, such as in teaching with students as beneficiaries and the value of inspiration, it is more likely to be satisfying for individuals. Therefore, an individual's self-schema and expected beneficiary impact must be aligned to achieve fulfillment. This involves performing a job that one enjoys, and that impacts the right beneficiary. For instance, Girschik et al. (2022) argued that activists serve specific beneficiary that they themselves choose, and therefore obtain their own perspective of what is socially responsible, compared to an organizational construction (i.e., “CSR”).

### 3.4. Utility value

Eccles and Wigfield (2020) describe the concept of utility value as the degree to which a particular task aligns with an individual's present or future plans and can be seen as a form of extrinsic motivation. Utility value can reflect significant goals that individuals deeply value, such as achieving a specific occupation. Although the distinction between utility and attainment is not always clear, our approach to utility value considers the utility for oneself *or* others.

#### 3.4.1. Self- and other utility

*One* way to conceptualize utility value is in terms of the benefits it provides for either the individual or others. Utility can be self-oriented, as when contributing to society leads to career advancement and goal achievement (Rosso et al., 2010), or it can be other-oriented. Examples of the latter are when the work impacts customers, human wellbeing, and the environment (Lips-Wiersma and Wright, 2012) or contributing to fighting diseases, political change, and environmental preservation (Martela and Pessi, 2018). Additionally, the utility value can extend to various groups, including coworkers, leaders, and family, or it can be for the betterment of society as a whole (Hall and Chandler, 2005; Elangovan et al., 2010; Hunter et al., 2010; Rosso et al., 2010). Thus, utility value encompasses both personal and societal goals.

#### 3.4.2. Actual utility

The degree of utility that individuals perceive as “enough” is dependent on their self-schemata. Individuals who are less interested in the beneficiary may be more self-oriented and less inclined to set high expectations for utility for others. In contrast, individuals who perceive their work as a calling may hold deeply rooted beliefs that the people affected by their work should benefit from it (e.g., Raatikainen, 1997; Bunderson and Thompson, 2009). The literature on meaningfulness often describes utility in terms of the impact vis-à-vis others, such as in the case of the work of Spinoza, Mother Theresa, or Mandela that is considered most meaningful (Metz, 2013). Michaelson (2021) and Ciulla (2012) argue that several values are more important for something to count as meaningful, leading to a “transcendental” perspective where values are not only part of the self-schemata, but also “out-there” or God-given. Such concept might apply to more religious self-schemata's (see Steger et al., 2010; Duffy and Sedlacek, 2011). However, this perspective may not always apply, particularly in a secular and individualistic world where different individuals may reason differently on which values are most important (Tyssedal, 2022). Therefore, contemporary employees might not desire to save the world, but incrementally make the world a better place, especially when a such contribution is beneficial for the self.

#### 3.4.3. Job possibilities to contribute

Nonetheless, socially responsible work is somewhat normative and shaped by institutional factors (see e.g., Michaelson, 2021). For instance, public policy work is generally seen as altruistic work (e.g., Perry and Wise, 1990; Ritz et al., 2020), whereas jobs related to financing and accounting may be perceived as less socially useful (Dur and Van Lent, 2019; Wolfe and Patel, 2019). Jobs related to healthcare, such as doctors and nurses, have gained greater public interest and recognition due to their roles in the COVID-19 pandemic (Kramer and Kramer, 2020). Additionally, advances in technology have impacted the meaning of work and its social value. The increasing use of big data, machine learning, and robotics have resulted in the automation of many jobs, leading to a loss of meaning and purpose in work for those who have been displaced (Kim and Scheller-Wolf, 2019). Others reported levels of societal meaninglessness, for example when employees feel alienated from policies, or that the job has an occupational stigma (Tummers et al., 2012; Shantz and Booth, 2014). Even within healthcare, (male) nurses may face negative perceptions from the media and the public, which can impact their sense of meaning and purpose in their work (Takase et al., 2006; Hoeve et al., 2014). The demands placed on individuals by society can vary depending on their personal interests and beliefs. This is particularly evident in individuals with an activist background who fight for societal or environmental causes. These individuals possess deeply rooted beliefs that may not align with the social responsibility policies of their employing organizations (Maks-Solomon and Drewry, 2021; Girschik et al., 2022; Reitz and Higgins, 2022). Their activism can take the form of protesting or strongly advocating for a particular social cause. As a result, they may not place much value on minor contributions. Consequently, such employees expect a specific outcome, to a specific extent, for the beneficiary, and evaluate it based not only on their self-schemata and opportunities but also on their interpretation of the institutional beliefs. As they have more knowledge on ecological values, they will more actively live in accordance with those roles, just like a religious person would live more aligned with the letter of the Bible.

### 3.5. Cost

Eccles-Parsons et al. (1983) asserted that individuals evaluate the costs and benefits of each activity or task and tend to avoid tasks with high costs relative to benefits, especially when compared to alternative tasks that offer a higher benefit-to-cost ratio (Eccles and Wigfield, 2020). The authors initially identified three types of costs, namely effort, opportunity, and emotional cost. The level of effort and opportunity costs incurred by employees are often dependent on the opportunities available in their job. Job opportunities that allow for social connections within and outside the organization enable employees to contribute to others and improve job satisfaction (e.g., Morgeson and Humphrey, 2006; Grant, 2007). However, as discussed before, not all jobs might be inherently including a socially responsible element. Hence, the job must, to a certain extent, allow employees to contribute to others. An effective corporate social responsibility (CSR) program can enhance employees' perception that contributing to society is worthwhile, as the organization emphasizes it (e.g., Aguinis and Glavas, 2019). In cases in which the job does not allow such opportunities, employees could engage in crafting to make their job more useful to society (Wrzesniewski and Dutton, 2001; Müller et al., 2019) or engage in extra-role behaviors that contribute (Seivwright and Unsworth, 2016). Engaging in such extra-roles might be demanding, leading to the expectation of additional costs that might lead to the belief that some socially responsible tasks are not worthy. Finally, there may be emotional costs involved in job engagement. Employees with a calling are more likely to experience emotional attachment to their job, leading to higher levels of burnout, but they are also able to control their obsessive passion since they consider their job important (e.g., Bunderson and Thompson, 2009; Schabram and Maitlis, 2017; Hirschi et al., 2019; Girschik et al., 2022). Therefore, contribution to society can be a double-edged sword in items of its implications.

**Figure 1:**
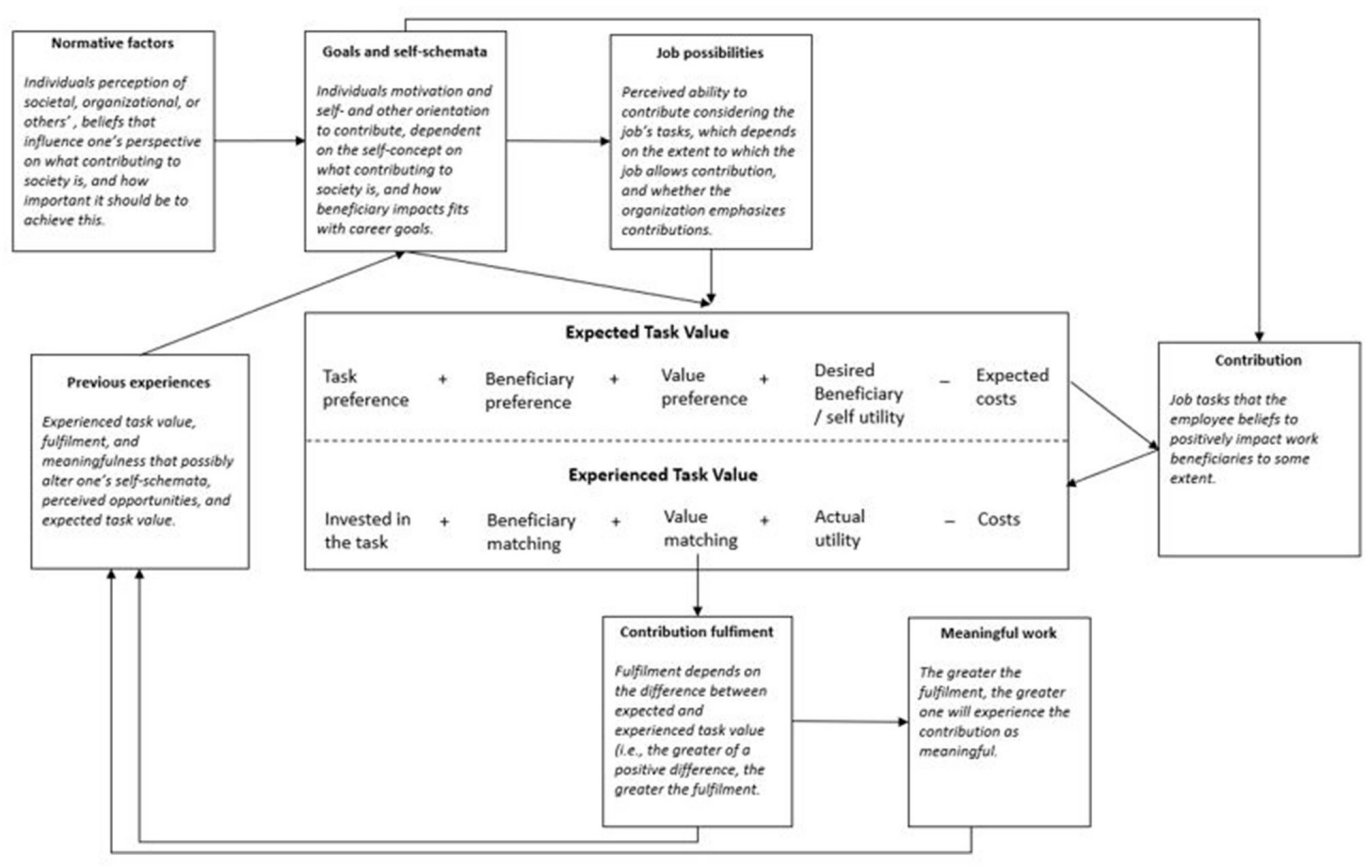
Contributing to society: a theoretical framework.

We can summarize this paragraph in Figure 1, in which we have developed an SEVT framework to better understand how individuals come to belief that what they do is contributing to society, and how individual preferences on (i) the task; (ii) the beneficiary; (iii) the value of the impact; (iv) desired utility; (v) perceived costs, strongly influence whether the contribution will be fulfilling.

## 4. Discussion

### 4.1. Conclusion

In this article, we explored the conceptualization of contributing to society, drawing on the calling and meaningful work literatures. While the conceptualizations progressed from an other-oriented perspective, the emphasis shift to self-orientation (i.e., seeking and finding fulfillment) (e.g., Dik et al., 2012; Lips-Wiersma and Wright, 2012; Lepisto and Pratt, 2017; Shimizu et al., 2019; Michaelson, 2021). Based on that literature, we have defined contributing to society as the belief that one's work should have a positive impact on, or is positively impacting beneficiaries, and highlighted the importance of considering individuals' reasons for perceiving something as a contribution. To better understand the complex relationship between self- and other-oriented reasons, we integrated Situated Expectancy-Value Theory (SEVT) with contributing to society. Our conceptual framework identified five key factors that influence an individual's fulfillment in contributing to society:

(i) Being invested in the task (i.e., contributing but pushing a button all day long, might not be very fulfilling);
(ii) Matching the beneficiary with personal preference (i.e., contributing to the natural environment, while someone might rather contribute to clients, is also not very fulfilling),
(iii) Matching the value of the impact with personal preference (i.e., contributing to clients by delivering them ecological value, while someone rather delivers innovative value, is also not very fulfilling),
(iv) The actual utility should match an individual's self- and other-oriented expectations of the outcome (i.e., someone that desires to climb the corporate ladder to contribute might desire limited beneficiary impact, compared to someone with a desire to achieve high beneficiary impact).
(v) The costs of engaging in tasks in which the individual believes to be contributing to society (i.e., if it is expected that the person will have to take additional roles, or roles that are difficult to reach, the costs become too high to be fulfilling).

Overall, our findings suggest that the relationship between meaningful work, calling, and contributing to society is complex, and requires a nuanced understanding of individual motivations and preferences. The level of fulfillment will depend on the expected contribution. While someone without a calling might be satisfied with a small contribution, someone that has high expectations will desire much higher levels of impact and is more committed to contributing (thereby also accepting potential costs).

### 4.2. Research agenda for contributing to society, calling, and meaningful work

Firstly, we should better understand contributing to society. While beliefs about contributions are dependent on the sense-making of that behavior (Wrzesniewski et al., 2003; Aguinis and Glavas, 2019), we have an insufficient understanding of how employees come to realize that their own work is contributing to beneficiaries (Bailey et al., 2019). As different individuals can have different sense-making processes for contributing is in their work, this could differ from an organizational process (Aguinis and Glavas, 2019; Girschik et al., 2022; Janssen et al., 2022). Qualitative work is useful. For instance, Silverman (2020) argued that sense-making is integral to being, and that all we value, whether egoistic or altruistic, stems from our personhood as embodied, embedded, enacted, and extended. Furthermore, are there any differences between jobs and sectors? According to several studies, some jobs, especially for-profit jobs, simply have limited opportunities to contribute to beneficiaries (Dur and Van Lent, 2019; Wolfe and Patel, 2019). How would employees make sense of a mismatch of the expected task value compared to the actual task value? And how do such employees make sense of parts of their work that are contributing compared to their entire job? It is imperative to gain a deeper understanding of the diverse interpretations and implications of “contributing to society” for individuals spanning various sectors, occupations, and cultural backgrounds. Nevertheless, this study is constrained by the fact that the existing literature predominantly comprises of theoretical expositions and empirical investigations that explore the role of contributing to society as a constituent of larger constructs such as meaningful work and calling. Given the salience of contributing to society in contemporary work scenarios, it would be beneficial to extend research efforts beyond the purview of these constructs and examine this phenomenon in isolation and study it as a stand-alone phenomenon.

Another critical question is how to map different beneficiaries and values that individuals desire and expect to contribute to. El Akremi et al. (2018) have developed a scale to measure organizational impact on various beneficiaries, which could be adapted to measure individual-level contributions. Additionally, policy capturing techniques may be useful for capturing the degree to which different individuals are likely to contribute to specific beneficiaries. Is a universal approach feasible, considering that individuals may not desire to contribute to multiple beneficiaries? Or are there beneficiaries that are generally more important? Furthermore, it is fruitful to map types of individuals to different values using justification frameworks, or “orders of worth,” which consist of moral narratives and objects that enable tests of worth. Boltanski and Thevenot (2006) have identified such orders in which each revolves around a specific set of values that are “worthy”, including inspired, domestic, civic, fame, market, and industry value. Bailey et al. (2019) proposed that these orders, that includes different values that employees could use to justify their work with regard to others that value from the same order, could lead to a better understanding of the role of the other in the experience of meaningfulness. While our perspective reinforces such claim, we do suggest that the orders should be interpreted as *preferences*. Where do these preferences come from? To what extent are they reinforced by the environment of that individual (i.e., is normative)? We argue that a better understanding on contributing to society could solve some of the conceptual issues and paradoxes that we have touched upon in earlier stages of this article.

Next, future studies should investigate the relationship between fulfillment and contribution to others, instead of juxtaposing the two dimensions. The meaning of life literature has found that for something to be deemed meaningful, it does not matter whether it is fulfilling or contributing to others (Prinzing et al., 2022). Replicating this research in the work environment is fruitful to get a better understanding if contributing should also be fulfilling. Is a contribution more fulfilling when the beneficiary matches; and how important is it that the value matches? And what if there is much contribution, but to a wrong beneficiary? Moreover, if fulfillment is indeed an important dimension in contributing to society, how, then, would we study calling, meaningful work, and their relationship? While the two dimensions are commonly juxtaposed (Bunderson and Thompson, 2009; Dik et al., 2012; Lips-Wiersma and Wright, 2012; Lepisto and Pratt, 2017; Shimizu et al., 2019; Michaelson, 2021), we propose that self- and other-orientation, and self-realization and contribution to others can be the same thing: employees get fulfilled when they contribute to others. Therefore, dependent on their own self-schemata, influenced by their stable characteristics, and contextual, normative, influences, the employee will develop preferences that include which and to what degree they desire to contribute to beneficiaries, values, and through what tasks. Therefore, future studies that conceptualize or operationalize meaningful and calling should be aware of this idea that self- and other-orientation are much more of a continuum. Finally, rather than a linear or moderating effect (Hirschi, 2011; see e.g., Duffy et al., 2012), calling and meaningful work should be studied in more comprehensive ways.

Lastly, meaningful work and calling scholars should be aware of our definition of contributing to society in future studies. Such progress is needed, since currently many terms are being used. These terms also relate to the idea that contribution should be directed at “the greater good”, “save the world”, or morality (Wrzesniewski et al., 1997; e.g., Steger et al., 2012; Michaelson, 2021). As individuals place a different emphasis and can expect to contribute different values to a variety of beneficiaries, there is no “one size fits all”. Therefore, future studies could shed light on differences in individual meaningfulness when considering the individual need satisfaction related to contributing to society (see also Blustein et al., 2023).

### 4.3. Research agenda for related fields

The present study is restricted in scope as it concentrates solely on the concept of contributing to society as viewed through the lenses of meaningful work and calling. It is important to acknowledge that there are other fields of inquiry that approach this phenomenon from distinct vantage points, and their insights may offer further nuance and depth to our understanding. First, the job design literature emphasizes the importance of job characteristics and relational job design, which align employees' prosocial motivation with significant tasks aimed at doing good (Grant, 2007, 2008a,b; Oldham and Hackman, 2010). Martela and Pessi (2018) argued that significant work, which is defined as having a broader purpose and self-realization, is a dimension of meaningful work. Moreover, prosocial motivation has often been the name of the sub dimension of calling that is about social responsibility (see e.g., Dik and Duffy, 2009; Elangovan et al., 2010; Hunter et al., 2010; Hirschi, 2011; Praskova et al., 2015; Dik and Shimizu, 2019). Therefore, it would be interesting to investigate whether the SEVT model also applies to this literature and whether there are any differences compared to the theoretical framework of contributing to society based on meaningful work and calling research. For instance, it is argued that prosocialness is more related to beneficiaries with whom the employee has contact with and is less relevant to understand contributions to society related to e.g., societal challenges, or greater good (see e.g., Schott et al., 2019; Ritz et al., 2020).

Second, scholars from the public policy literature argue that employees have the Public Service Motivation (PSM) to contribute to the public sector (Perry and Wise, 1990; Perry, 1996). Recent studies have shown that PSM and calling, prosocial motivation, and task significance are complementary (Thompson and Christensen, 2018; Schott et al., 2019; Ritz et al., 2020; Vogel, 2020). One possible research avenue is to investigate whether the PSM, calling, and prosocial motivation, form a second-order construct of self-schemata. As all three constructs in one way or another, and to a different degree, emphasize the motivation to contribute to society, a collaboration could lead to a better understanding on why employees contribute to society.

## Author contributions

RB, EL, and SK contributed to conception and analysis of the study. RB wrote the first draft of the manuscript. EL and SK wrote sections of the manuscript. All authors contributed to manuscript revision, read, and approved the submitted version.

## Footnotes

^1^With self- and other-orientation we mean the tension between the need to meet the needs of others and that of the self (Lips-Wiersma and Wright, 2012). Therefore, the question is to what degree someone serves the self compared to serving others (e.g., Bailey et al., 2019; Michaelson and Tosti-Kharas, 2019).

^2^Contribution to society could be discussed from prosocial and public policy literature. However, we argue that it is best to discuss the topic from the calling and meaningful work literature. First, prosocial motivation and behavior emphasize impacting the organization and colleagues and does not cover the broader scope of potential beneficiaries that we consider as “society” (Schott et al., 2019; Piatak and Holt, 2020; Ritz et al., 2020). Second, public service and prosocial motivations are motivations that are about *actual* behaviors (see e.g., Perry, 1996; Grant, 2007). However, we will propose that contributing to society is rather a *motivated belief* : this means that different individuals will construct a different perspective on what is “society”, and what contributive behaviors are.

^3^As prosocial and public service motivation are about *actual* behaviors, a Theory of Planned Behavior (TPB) approach fits, as the main limitation of TPB is that it revolves around actual rather than interpretations of behaviors (Fishbein and Ajzen, 2010; Sniehotta et al., 2014). Additionally, TPB attitudes concern an individual's evaluation of the behavior, without considering expected outcomes such as fulfillment (Eccles and Wigfield, 2002). Therefore, as calling and meaningful work concern the interpretation of behavior, SEVT is a more viable framework.

^4^This approach is opposed to Corporate Social Responsibility (CSR). Unlike CSR, which centers around organizational decisions on beneficiaries and strategies (McWilliams et al., 2006; Sharp and Zaidman, 2010), contributing to society pertains to individual actions that the *individual* perceives as contributing to others. While these actions may align with the beneficiary and strategy chosen by the organization, they may also diverge from them.
